# Examining Comorbidity Patterns Among Prostate Cancer Patient-Partner Dyads

**DOI:** 10.1093/geroni/igaf122.3761

**Published:** 2025-12-31

**Authors:** Chunxuan Ma, Bianca Shieu, Lixin Song

**Affiliations:** Case Western Reserve University, Cleveland, Ohio, United States; University of Texas Health Science Center at San Antonio, San Antonio, Texas, United States; UT Health San Antonio, San Antonio, Texas, United States

## Abstract

**Background:**

Prostate cancer (PCa) patients and their partners both experiences reduced quality of life and challenges in maintaining well-being when comorbidities are present. This study aims to describe the pattern of comorbidities for PCa patients and partners.

**Methods:**

We defined comorbidity burden as the mean of the Charlson Comorbidity Index (CCI) score, and comorbidity congruence as the absolute difference in the CCI score within each dyad. Using the baseline data from a randomized clinical trial (RCT), we fitted multilevel models to examine how social determinants of health (SDOH) influenced comorbidity congruence. We used network analysis to identify comorbidity co-occurrent patterns.

**Results:**

Among 273 dyads in the RCT, 118 dyads lived in high Area Deprivation Index (ADI) ranking areas and 54 patients were Black. Dyads whose partner reported non-white and non-Black (N = 13) exhibited significantly greater discrepancies in comorbidity counts compared to whose partner was White or Black (p = 0.04). Dyads who lived in high ADI areas, who’s income >$90,000, or with higher mean age had significantly higher CCI burden (ps < 0.01). We observed co-occurrence of myocardial infarction–heart failure, chronic liver disease–stroke, and stroke–heart failure among patients. We observed myocardial infarction–heart failure and stroke–heart failure among partners.

**Conclusion:**

The overall comorbidity characteristics of PCa patient-partner dyads vary by SDOH and neighborhood deprivation. Cardiovascular and stroke comorbidity patterns differ between patients and partners. Understanding comorbidity from a dyadic perspective is essential for designing comprehensive, postoperative well-being interventions for PCa patients and partners, particularly in socioeconomically disadvantaged areas.

